# Utility of molecular studies in incontinentia pigmenti patients

**Published:** 2011-04

**Authors:** Seema Thakur, Ratna D. Puri, Sudha Kohli, Renu Saxena, I.C. Verma

**Affiliations:** *Department of Genetic Medicine, Sir Ganga Ram Hospital, New Delhi, India*

**Keywords:** Incontinentia pigmenti (IP), *NEMO* gene, skin lesions

## Abstract

The diagnosis of incontinentia pigmenti (IP) is fairly easy in the presence of classical features, but can be difficult in cases with partial or non-classical features, especially in the parents. The demonstration that the disease is caused by mutations in the *NEMO* gene, has remarkably improved genetic counselling for this disorder. We present four families of IP in whom molecular studies established an unequivocal diagnosis in the affected daughters, and showed two mothers to be carriers, thus allowing accurate genetic counselling and prenatal diagnosis.

Incontinentia pigmenti (IP; Bloch- Sulzberger syndrome; MIM 308300) is a rare genodermatosis that occurs in approximately 1 in 50,000 newborns^1^–^4^. It is an X- linked dominant disorder that affects females and is lethal *in utero* for males. It is a multi- system disorder primarily affecting ectodermal tissues such as skin, hair, nails, teeth, eyes and central nervous system. The skin lesions of IP evolve through four stages, but all the stages may not be seen in all cases. The disorder is caused by mutation in *NEMO* (NF - KB essential modulator) gene, which is located on Xq28. *NEMO* is a 23kb gene composed of 10 exons. Delta *NEMO* (nonfunctional second copy of the *NEMO* gene) is located 31.6 kb from exon 10. NF-Kb regulates immune and inflammatory response and is involved in prevention of apoptosis in response to TNF- alpha. Ectodermal dysplasia with immunodeficiency (ED-ID) and ectodermal dysplasia with immunodeficiency, osteopetrosis and lymphedema (OL-ED-ID) are allelic to IP as both are caused by mutation in the same gene (*NEMO*)^5^. The mutation causing IP are severe compared to those causing ED-ID and OL- ED-ID. Mutation analysis of *NEMO* gene confirms the clinical diagnosis and is necessary for prenatal diagnosis in future pregnancies. The study was done on four consecutive IP patients who presented to us for genetic counselling or prenatal diagnosis during 2005-2008.

**Case 1:** A 33-year-old second gravida consulted at 7 weeks of pregnancy. Her first child was a 12 yr old daughter who had short stature (height 142 cm), with hypopigmented skin lesions along the Blaschko lines all over the body. Blisters were present at birth. Biopsy of these lesions showed different stages of skin lesions consistent with incontinentia pigmenti. There was no vision in the left eye due to retinal detachment at the age of 1.5 yr. Nails were dystrophic in both hands and feet. She had partial anodontia- total teeth 18. The mother had short stature and she also had partial anodontia with minimal skin lesions. Her nails and hair were normal with no eye lesions. Both mother and daughter did not have any CNS manifestation.

The grandmother of index case was also short and had abnormal teeth. There was history of death of male children soon after birth in grandmother’s sister. Molecular studies confirmed the diagnosis in both mother and her daughter and prenatal diagnosis was provided in her ongoing pregnancy.

**Case 2 :** A 2 yr old girl with hypopigmented skin lesions had blisters present at the time of birth. Skin biopsy at day 9 suggested IP. Her eyes, teeth and nails were normal. There was no mental retardation. Examination of parents for any skin lesions, nail or hair changes was negative. There was no history of other affected females or recurrent foetal losses. Molecular studies established the diagnosis in the affected child, whereas the mother was shown not to be a carrier.

**Case 3:** A 4 yr old girl born to nonconsanguineous parents, had hyperpigmented skin lesions along Blaschko lines at the time of presentation (Fig. 1). She had blisters present at the age of one week. Skin biopsy suggested stage 2 of IP. She had oligodontia. Her eyes and nails were normal. Examination of parents for any skin lesions, nail or hair changes was negative. Molecular studies confirmed the diagnosis in the daughter, whereas the mother was excluded as a carrier.

**Fig. 1:**
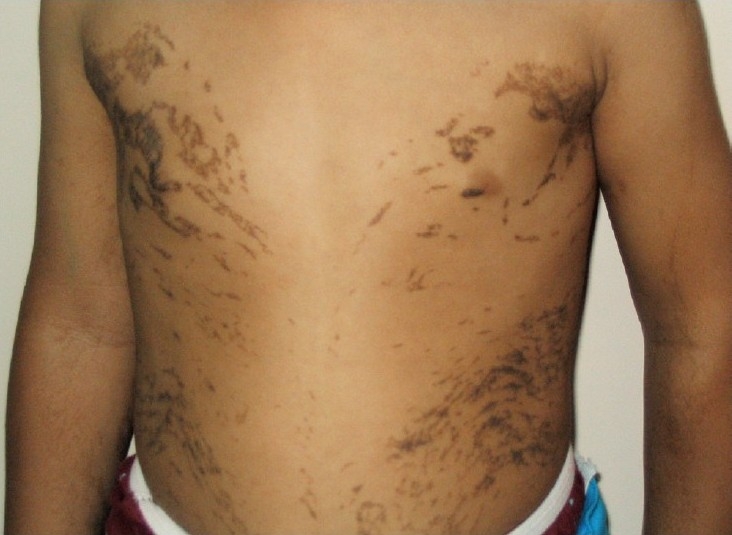
Case 3- Skin lesions at hyperpigmented stage.

**Case 4:** This family had come for prenatal diagnosis of IP as their 5 yr old daughter was clinically suspected to have IP. She had hypopigmented streaks in whorled pattern but not along Blaschko lines. These hypopigmented lesions were preceded by rough papules. There was no history of vesicles at birth. The child had peg- shaped teeth. She had history of seizures. Her mother had atypical hypopigmented skin lesions. Both mother and daughter had the deletion of *NEMO* gene thus confirming the diagnosis.

Clinical features of cases are summarized in the Table.

**Table:** Clinical characteristics of cases with incontinentia pigmenti

Case No.	Age (yr)	Age at diagnosis	Typical skin lesions	Teeth	Hair and nails	Eye lesions	Mental retardation	Parents
Case 1	12	At birth	+	+	+	+	-	Mother-affected
Case 2	2	At birth	+	-	-	-	-	Normal
Case 3	4	At birth	+	+	-	-	-	Normal
Case 4	5	2.5 yr	No blisters	+	-	-	Seizures	Mother affected

*Molecular diagnosis of incontinentia pigmenti:* DNA was isolated from the peripheral blood from all affected girls and their parents. The deletion in *NEMO* gene was analyzed by two PCR reactions according to the protocol described earlier^6^,^7^. Multiplex PCR as described by Steffann *et al*^6^ was done first to detect deletion in *NEMO* or pseudogene. In the method, the internal control of amplification is present which rules out any PCR artifact when rearrangement of *NEMO* or pseudogene is absent, but this test cannot discriminate between *NEMO* and psudogene rearrangement. So, the PCR method described by Bardaro, *et al*^7^ to distinguish between *NEMO* and psudogene rearrangement was done.

PCR showed that the index case in all the families had a band corresponding to the deletion of exon 4-10 in *NEMO* gene (1045 bp; Fig. 2). The mother in the cases 1 and 4 also had the same deletion whereas parents were normal in other two families.

**Fig. 2:**
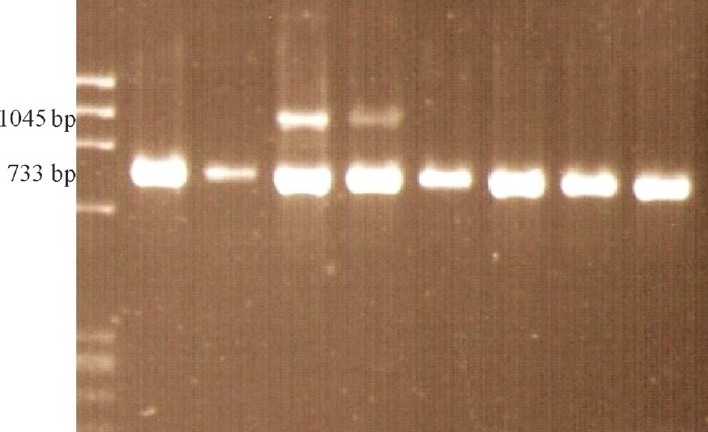
Multiplex PCR products in IP patients and controls. Presence of 1045 bp band indicates the presence of the common rearrangement in IP patients only. 733 bp product serves as internal amplification control. Lane 1: ΦX174 DNA/ (HaeIII digested) DNA marker; Lane 2: Mother of case 2; Lane 3: Father of case 2; Lane 4: Case 2, Positive for deletion; Lane 5: Case 3, Positive for deletion; Lane 6: Mother of case 3; Lane 7: Father of case 3; Lane 8 & 9: Normal control (As this is a representative picture and the cases were analyzed at different times, the mothers of other cases are not shown).

Diagnosis of IP is made by the diagnostic criterion given by Landy and Donai^2^. Significant clinical heterogeneity exists in IP with regard to ectodermal, ophthalmologic, and neurologic abnormalities, even within families. Skin abnormalities are consistent features and usually occur in four stages. Erythema and blistering of skin occur in a linear distribution (Blaschko lines) along the limbs and around the trunk within the first few weeks of life (Stage 1). The vesicular stage has been reported to occur in 90-95 per cent of patients. In most patients (>90%), lesions are present at birth or develop within the first 2 weeks of life. Hyperkeratotic lesions follow this on the limbs or trunk (Stage 2). Verrucous lesions have been reported to occur in 70-80 per cent of IP patients. In most patients, verrucous lesions develop in the first few weeks to months of life and subsequently resolve over weeks to months. Hyperpigmented lesions develop, most apparent on trunk and occur in streaks or whorls in next stage. (Stage 3). Hyperpigmented lesions generally develop within the first few months of life and resolve slowly by adolescence. Pale hairless patches most evident on the lower legs characterize stage 4. All our cases had skin lesions. However, case 4 had skin lesions which were not classic of IP. She did not have vesicles at birth. Dental abnormalities include hypodontia, delayed eruption or conical form. Dental abnormalities are seen in 80 per cent of patients and can involve both deciduous and permanent teeth. Three of our cases had hypodontia. Hair changes include scarring alopecia and are seen in 28-38 per cent of patients. Only case 1 had thin hair. Nail features include nail dystrophy, which ranges from mild pitting or ridging of the nail plate to hyperkeratosis and onycholysis. This is observed in 7-40 per cent of IP patients, and usually multiple fingernails and toenails are affected^2^,^8^. Only case 1 had nail dystrophy. Ophthalmologic findings occur in 0-77 per cent of patients, and asymmetric involvement is common. The spectrum of changes that have been reported are nystagmus, strabismus, microphthalmos, ptosis, blue sclera, pigmentation of the conjunctiva, corneal changes, cataract, optic atrophy, vitreous haemorrhage and myopia. However, the most typical abnormality is fibroblastic retinal detachment secondary to an ischaemic Vasculopathy^9^. Case 1 had retinal detachment and had no vision in left eye.

Approximately one third of the patients have mental deficiency. All affected girls did not have mental retardation. Case 4 had seizures. Carney^8^ observed that about 30 per cent have notable CNS disease, however, Landy and Donnai found much lower incidence (10%)^2^.

IP is familial in 10-25 per cent of cases. In this study, two cases had affected mother whereas parents were normal in the remaining two cases. IP is an X- linked dominant disorder and the risk of recurrence depends upon mutation status of parents. If parents have a deletion in the *NEMO* gene, risk is 50 per cent to all offsprings (male and females). Affected male children do not survive beyond second trimester. If parents do not have deletion the risk in next pregnancy is low (about 1%).

Multiplex PCR of the *NEMO* gene was done first which showed 1045 bp band in all four cases. This confirmed the clinical diagnosis in all the four cases. By this multiplex PCR, rearrangement of both *NEMO* gene and pseudogene is detected. To exclude pseudogene rearrangement PCR method described by Bardaro, *et al*^7^ was done in all cases. All four cases had deletion in *NEMO* gene. These tests detect the common rearrangement as recurrent exon 4-10 genomic rearrangement in the *NEMO* gene accounts for 60 to 80 per cent of IP-causing mutations^10^,^11^.

Prenatal as well as postnatal diagnosis is best done by the combined use of molecular tests^6^,^7^ to detect the deletion and direct sequencing of the gene, if deletion is absent.

Most of the studies on IP are reported from western countries^12^. A few reports of IP are available from China, Korea and Japan^12^. In general, the frequency of *NEMO* mutation is similar^12^. Besides the *NEMO* rearrangement found in IP females (which is lethal in males), a total of 69 different small mutations (missense, frameshift, nonsense, and splice-site mutations) have been reported, including 13 novel ones^11^. Hence, if deletion is absent sequencing of *NEMO* gene would be required. X-inactivation studies may help further.

In conclusion, clinical features of IP are variable and difficult to diagnose in cases with mild manifestations. Molecular diagnosis may help confirm the clinical suspicion and is essential for providing definite genetic counselling and prenatal diagnosis.

## References

[1] Goldberg MF, Custis PH (1993). Retinal and other manifestations of incontinentia pigmenti (Bloch Sulzberger syndrome). *Opthalmology*.

[2] Landy SJ, Donnai D (1993). Incontinentia pigmenti (Bloch Sulzberger syndrome). *J Med Genet*.

[3] Cohen PR (1994). Incontinentia pigmenti: Clinicopathological characteristics and differential diagnosis. *Cutis*.

[4] Cohen PR, Kurzrock R (1995). Miscellaneous genodermatoses: Beckwith Wiedmann syndrome, - Hogg -Dube syndrome, familial atypical multiple mole melanoma syndrome, hereditary tylosis, incontinentia pigmenti and supernumerary nipples. *Dermatol Clin*.

[5] Priolo M (2009). Ectodermal dysplasias: An overview and update of clinical and molecular-functional mechanisms. *Am J Med Genet*.

[6] Steffann J, Raclin V, Smahi A, Woffendin H, Munnich A, Kenwirick SJ (2004). A novel PCR approach for prenatal detection of the common NEMO rearrangement in incontinentia pigmenti. *Prenat Diagn*.

[7] Bardaro T, Falco G, Sparago A, Mercadante V, Molins EG, Tarantino E (2002). Two cases of misinterpretation of molecular results in incontinentia pigmenti and a PCR based method to discriminate *NEMO/IKKY* gene deletion. *Human Mut*.

[8] Carney RG (1976). Incontinentia pigmenti: a world statistical analysis. *Arch Dermatol*.

[9] Doherty MO, Creery KMC, Green AJ, Tuwir I, Brosnahan D (2011). Incontinentia pigmentidophthalmological observation of a series of cases and review of the literature. *Br J Ophthalmol*.

[10] Aradhya S, Woffendin H, Jackins T, Bardaro T, Esposito T, Smahi A (2001). A recurrent deletion in the ubiquitously expressed *NEMO (IKK-Y)* gene accounts for the vast majority of Incontinentia pigmenti mutations. *Hum Mol Genet*.

[11] Fusco F, Pescatore A, Bal E, Ghoul A, Paciolla M, Lioi MB (2008). Alterations of the IKBKG locus and diseases: An update and a report of 13 novel mutations. *Hum Mutat*.

[12] Hsiao PF, Lin SP, Chiang SS, Wu YH, Chen HC, Lin YC (2010). *NEMO* gene mutations in Chinese patients with incontinentia pigmenti. *J Formos Med Assoc*.

